# iTRAQ-BASED Proteomic Analysis of the Mechanism of Fructose on Improving Fengycin Biosynthesis in *Bacillus Amyloliquefaciens*

**DOI:** 10.3390/molecules26206309

**Published:** 2021-10-19

**Authors:** Hedong Lu, Ruili Li, Panping Yang, Weibo Luo, Shunxian Chen, Muhammad Bilal, Hai Xu, Chengyuan Gu, Shuai Liu, Yuping Zhao, Chengxin Geng, Li Zhao

**Affiliations:** 1 School of Life Science and Food Engineering, Huaiyin Institute of Technology, Huaian 223003, China; luhd100@hyit.edu.cn (H.L.); climbing123@163.com (P.Y.); bilaluaf@hotmail.com (M.B.); sliverhai@163.com (H.X.); guchengyuan202108@163.com (C.G.); liushuai007@hyit.edu.cn (S.L.); zhaoyuping@hyit.edu.cn (Y.Z.); 2 College of Food Science and Technology, Fujian Agriculture and Forestry University, Fuzhou 250003, China; L19959211086@163.com; 3 Institute of Food and Marine Bio-Resources, College of Biological Science and Technology, Fuzhou University, Fuzhou 350108, China; nxzgcs888@163.com (W.L.); 15659209187@163.com (S.C.)

**Keywords:** *Bacillus amyloliquefaciens*, fengycin, fructose, mechanism, iTRAQ

## Abstract

Fengycin, as a lipopeptide produced by *Bacillus subtilis*, displays potent activity against filamentous fungi, including *Aspergillus flavus* and *Soft-rot fungus*, which exhibits a wide range of potential applications in food industries, agriculture, and medicine. To better clarify the regulatory mechanism of fructose on fengycin biosynthesis, the iTRAQ-based proteomic analysis was utilized to investigate the differentially expressed proteins of *B. amyloliquefaciens* fmb-60 cultivated in ML (without fructose) and MLF (with fructose) medium. The results indicated that a total of 811 proteins, including 248 proteins with differential expression levels (162 which were upregulated (fold > 2) and 86, which were downregulated (fold < 0.5) were detected, and most of the proteins are associated with cellular metabolism, biosynthesis, and biological regulation process. Moreover, the target genes’ relative expression was conducted using quantitative real-time PCR to validate the proteomic analysis results. Based on the results of proteome analysis, the supposed pathways of fructose enhancing fengycin biosynthesis in *B. amyloliquefaciens* fmb-60 can be summarized as improvement of the metabolic process, including cellular amino acid and amide, fatty acid biosynthesis, peptide and protein, nucleotide and nucleobase-containing compound, drug/toxin, cofactor, and vitamin; reinforcement of peptide/protein translation, modification, biological process, and response to a stimulus. In conclusion, this study represents a comprehensive and systematic investigation of the fructose mechanism on improving fengycin biosynthesis in *B. amyloliquefaciens*, which will provide a road map to facilitate the potential application of fengycin or its homolog in defending against filamentous fungi.

## 1. Introduction

Fengycin is a cyclic lipopeptide (CLP) composed of a β-hydroxy fatty acyl chain and a decapeptide with eight kinds of amino acids. It displays potent and broad-spectrum activities against filamentous fungi [1]. Recently, a great interest has been boosted in developing natural antimicrobials due to increasing demands for minimizing the application of chemical preservatives in the processed food [2]. *Bacillus amyloliquefaciens*, as a Gram-positive bacteria, is granted as GRAS (generally recognized as safe) strain in the food and feed industry [3]. *B. amyloliquefaciens* can produce a number of antimicrobial compounds, consisting of surfactin, fengycin and bacillomycin D [4,5]. Among these, fengycin displays potent antimicrobial activity against foodborne filamentous fungi, such as *Aspergillus flavus*, *Soft-rot fungus* and *Fusarium moniliforme*, which exhibit potential applications in agriculture, biological control, food and feed industry [6,7]. For instance, fengycin and other antimicrobial lipopeptides produced by *B. amyloliquefaciens* ES-2 can remarkably reduce the growth of *Shewanella putrefaciens* in shrimp meat [8]. Similarly, some reports have shown that fengycin can induce apoptosis and necrosis of *Rhizopus stolonifer*, indicating its potentiality in food preservatives [9]. Furthermore, the growth of cancer cell line 95 D is inhibited, and cell apoptosis is promoted after being exposed to fengycin [10]. The lipopeptide has been widely used in agriculture to control plant pathogenic, including *Fusarium verticillioides*, *Fusarium moniliforme* and *Botrytis cinerea* [11,12,13].

Fengycin is produced by multi-modular proteins called non-ribosomal peptide synthetases (NRPSs) consisted of five multifunctional enzymes: FenA, FenB, FenC, FenD and FenE, which containten activating sites and a cyclase module. These multifunctional enzymes catalyze ten amino acids into a cyclic decapeptide; then fatty acyl chain is attached to the peptide. Numerous genes, including *codY*, *comA*, *degU* and *spo0A* are also involved in regulating the biosynthesis of fengycin [14]. In previous works, *B.amyloliquefaciens* fmb-60 is able to enhance the yield of fengycin and upregulate the gene expression of *fen* in a modified Landy medium supplemented with fructose [15], but the regulatory mechanism of fructose on fengycin biosynthesis has not been elucidated yet. In this study, iTRAQ-based proteomic analysis was utilized to explain the mechanism of fructose on fengycin biosynthesis. The results provided new insights into better explicating the signaling network between fructose and fengycin biosynthesis and provide a theoretical basis for further fengycin production improvement.

## 2. Results

### 2.1. Effect of Fructose on the Yield of Fengycin 

**Figure 1:**
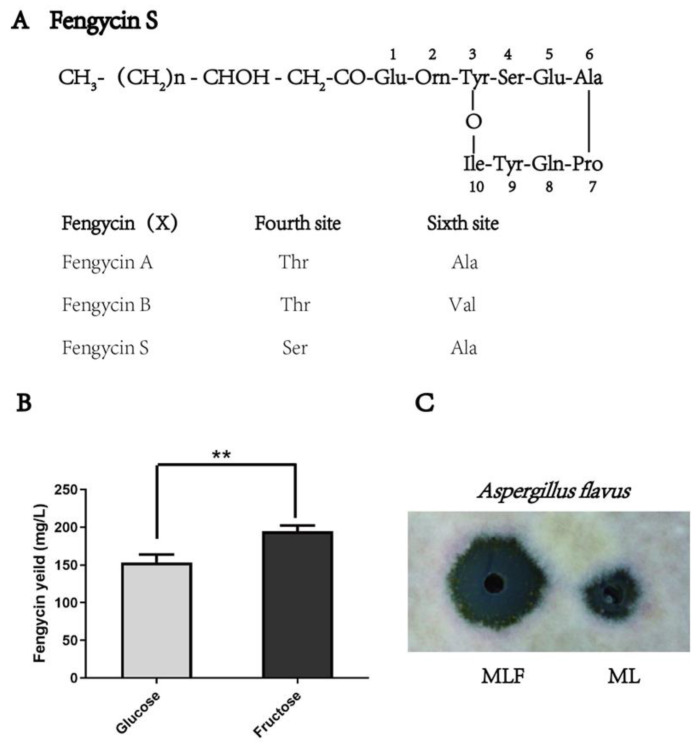
(**A**) The molecular structure of fengycins (**B**) The effort of fructose on the yield of fengycin ** means significant differences (**C**) The antimicrobial activity of the culture supernatant of ML and MLF against *Aspergillus flavus* after being fermented by *B. amyloliquefaciens* fmb-60.

The inclusion of fructose was found to be useful to increase the yield of fengycin(Figure 1A). As shown in Figure 1B, a higher yield of fengycin was determined in the culture supernatant, when *B. amyloliquefaciens* was cultivated in the modified Landy medium supplemented with fructose as the carbon resource (MLF). Moreover, the culture supernatant of MLF also exhibited more potent antimicrobial activity against *Aspergillus flavus* after being fermented by *B. amyloliquefaciens* (Figure 1C).

### 2.2. Analysis of Primary Data and Identification of Protein 

**Figure 2:**
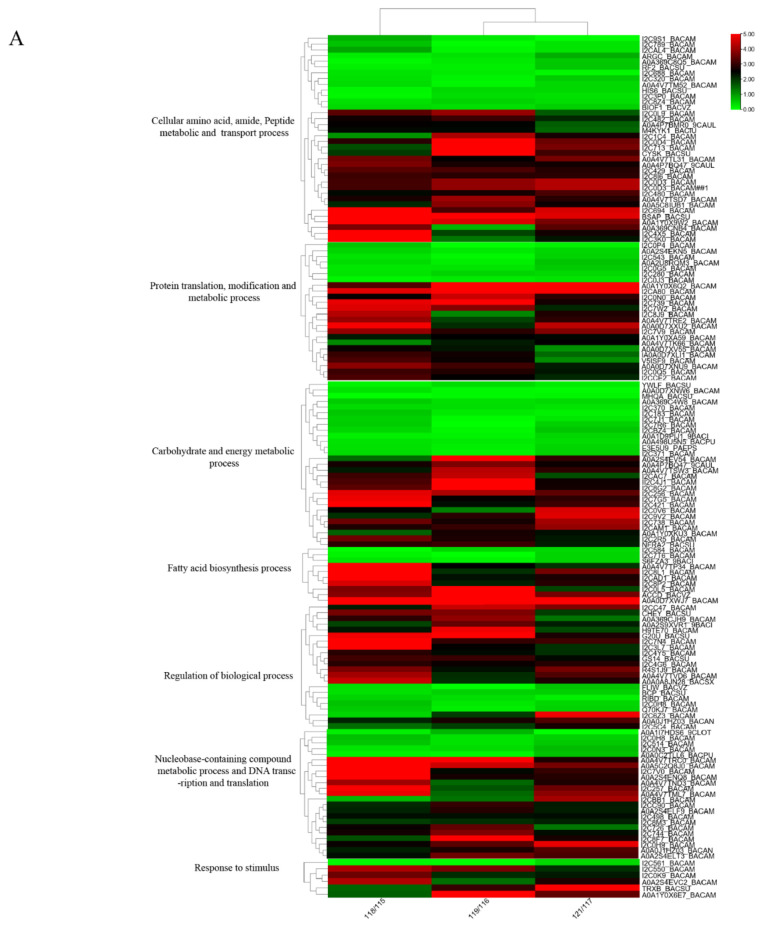

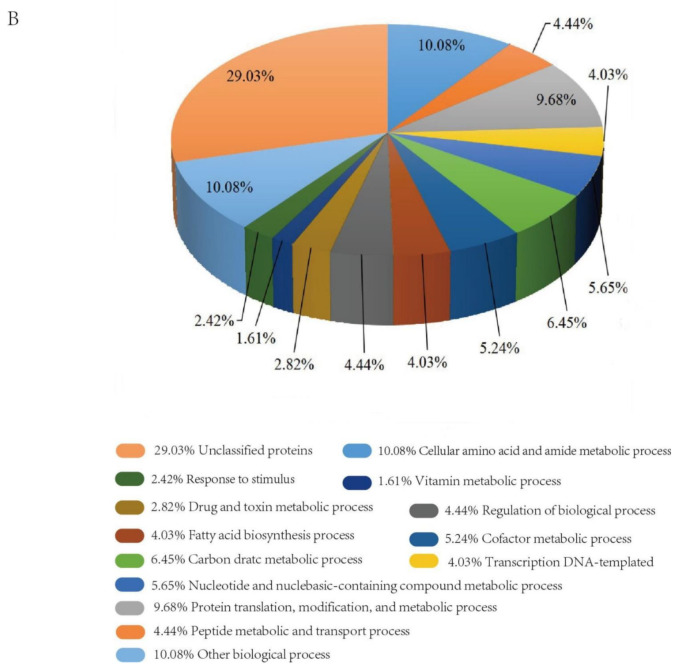
(**A**) Heat map of protein expression. (**B**) Gene ontology (GO) term enriched broader process classification of the 248 differentially expressed proteins during *B. amyloliquefaciens* fmb-60 cells cultivated in ML and MLF.

The regulation of gene expression can occur at different levels, such as transcription and translation. Proteomic analyses were carried out to scrutinize the changes on protein level during the biosynthesis process of fengycin. According to the protein abundance level, a total of 811 proteins were identified during the computational following iTRAQ-MS analysis of cell extracts. In addition, when protein expression multiple folds of *B. amyloliquefaciens* cells cultivated in MLF medium compared to the counterparts in ML medium are greater than to 2 times (up-regulated) or less than 0.5 times (down-regulated), and the statistical test of *p*-value is less than 0.05, 248 differentially expressed proteins was identified including of 162 up-regulated and 86 down-regulated expressed-proteins. The Cluster analysis was performed based on the data of iTRAQ-based proteomics (Figure 2A). In the classification of biological processes, differential proteins were mainly related to the cell and intracellular organics biosynthesis and metabolic process, including cellular amino acid, amide, peptide metabolic and transport process, fatty acid biosynthesis process, modification and metabolic process, carbohydrate and energy metabolic process, drug and toxin metabolic process, protein translation, and so on(Figure 2B). 

### 2.3. Functional Categories of Differentially Expressed Proteins

#### 2.3.1. Cellular Amino Acid, Amide, Peptide Metabolic and Transport Process 

**Figure 3:**
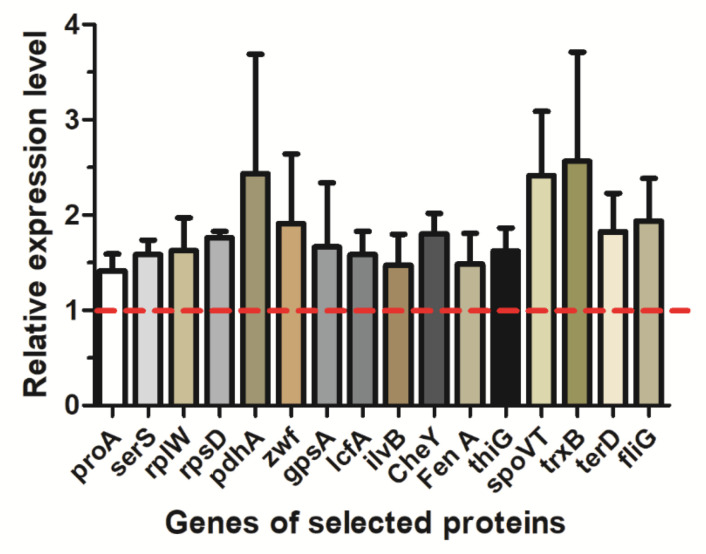
Relative expression levels of transcripts. Data were normalized against expression of the housekeeping gene gatB_Yqey. To determine relative fold differences for each gene, the formula 2^−^^ΔΔCt^ was used. ΔΔCT = ΔCT Treated group (CT (target gene) − CT (endogenuous reference gene)) − ΔCT control group (CT (target gene) − CT (endogenuous reference gene)).

Of the measured proteins, 36 displayed significant changes with abundance in the MLF medium (Table S1), which had significant changes in cellular amino acid, amide, peptide metabolic and transport process, including Gamma-glutamyl phosphate reductase, cysteine synthase, serine-tRNA ligase, cysteine-tRNA aspartokinase, V ligase, aline-tRNA ligase, aminopeptidase YwaD precursor, spermidine synthase, peptidase M42, dipeptidase PepV, probable cytosol aminopeptidase, oligoendopeptidase F, and peptide chain release factor 2. In addition, the results of qRT-PCR assays showed that the expression of Gamma-glutamyl phosphate reductase gene (*proA*) and Serine--tRNA ligase gene (*serS*) were upregulated, which were corresponding to proteomic analysis results (Figure 3). It was supposed that fructose could promote the amino acid, amide, peptide metabolic and transport process, resulting in fengycin accumulation in *B. amyloliquefaciens*.

#### 2.3.2. Protein Translation, Modification, and Metabolic Process

We identified 24 differentially expressed proteins, including 17 upregulated proteins and 7 downregulated ones (Table S1) involved in protein translation, modification, and metabolic process. Among them, 50S ribosomal protein L23, 50S ribosomal protein L13, 30S ribosomal protein S4, Lon protease, putative pyruvate and phosphate dikinase regulatory protein were upregulated (Table S1). Moreover, the results of qRT-PCR results indicated the expression level of 50S ribosomal protein L23 (*rplW*) and 30S ribosomal protein S4 (*rpsD*) were upregulated (Figure 3), which was consistent with the proteomic analysis results. It was inferred that the exposure to fructose promoted protein translation, modification, and metabolic process, contributing to fengycin biosynthesis.

#### 2.3.3. Carbohydrate and Energy Metabolic Process

Twenty-nine proteins displayed significant alterations involved in the carbohydrate and energy metabolic process. Among them, glucose-6-phosphate 1-dehydrogenase, glycerol-3-phosphate dehydrogenase (NAD(P)+), fructosamine deglycase, gluconokinase, 6-phosphogluconolactonase, FMN reductase (NAD(P)H)was upregulated, and glutamine--fructose-6-phosphate aminotransferase and acyl-CoA dehydrogenase was downregulated (Table S1), these are related to the metabolic process of fructose, as well as the biosynthesis and homeostasis of ATP, NAD(+)H and NADP(+)H. Additionally, qRT-PCR result showed the gene expressions of pyruvate dehydrogenase E1 component subunit alpha gene *(pdh A*), glucose-6-phosphate 1-dehydrogenase gene *(zwf)* and glycerol-3-phosphate dehydrogenase (NAD(P)+) gene (*gps A*) were up-regulated (Figure 3), which is consistent to the proteomic analysis results. It was indicated that fructose was possibly reformulating the carbohydrate metabolism and energy supplying in *B. amyloliquefaciens* cells, which may be conducive to synthesize fengycin. 

#### 2.3.4. Fatty Acid Biosynthesis Process

Fatty acid chains are one of the key components of fengycin. Therefore, the rate of the fatty acid biosynthesis process limits fengycin production. As showed in Table S1, ten differentially expressed proteins involved in the fatty acid biosynthesis process were identified. Seven proteins, including acetyl-coenzyme A carboxylase carboxyl transferase subunit beta, 2-C-methyl-D-erythritol 2,4-cyclodiphosphate synthase, lactate utilization protein B, acyl carrier protein, long-chain fatty-acid-CoA ligase, 3-hydroxy-3-methylglutaryl-ACP synthase and acetolactate synthase, were upregulated. Three proteins, containing1-deoxy-D-xylulose 5-phosphate reductoisomerase, 4-hydroxy-3-methylbut-2-en-1-yl diphosphate synthase (flavodoxin) and enoyl-CoA hydratase/3-hydroxy acyl-CoA dehydrogenase, were downregulated. qRT-PCR results suggested that long-chain fatty-acid-CoA ligase gene (*lcfA*) and acetolactate synthase gene *(ilvB)* expressions were all upregulated, in accord with the results of proteome analysis (Figure 3). The results indicated that the cultivation with fructose as the sole carbon source was conducive to fatty acid biosynthesis, enhancing fengycin yield.

#### 2.3.5. Regulation of Biological Process

Eleven differentially expressed proteins related to the regulation of biological processes were identified. Among them, eight proteins, including Chemotaxis protein CheY, General stress protein 14, DeoR family transcriptional regulator, Alkyl hydroperoxide reductase subunit F, Ribonuclease Y, Alkyl hydroperoxide reductase C, General stress protein 20 U and HTH-type transcriptional regulator MntR were upregulated, and three proteins including Flagellar assembly factor FliW, Peroxiredoxin Bcp and Transcription-repair-coupling factor were downregulated. qRT-PCR results showed that the Chemotaxis protein CheY gene (*CheY*) expression was upregulated/downregulated (Figure 3). It was indicated that exposure to fructose is helpful to enhance the capacity of biological regulation in *B. amyloliquefaciens* cells, which may contribute to fengycin biosynthesis.

#### 2.3.6. Drug and Toxin Metabolic Process

**Figure 4:**
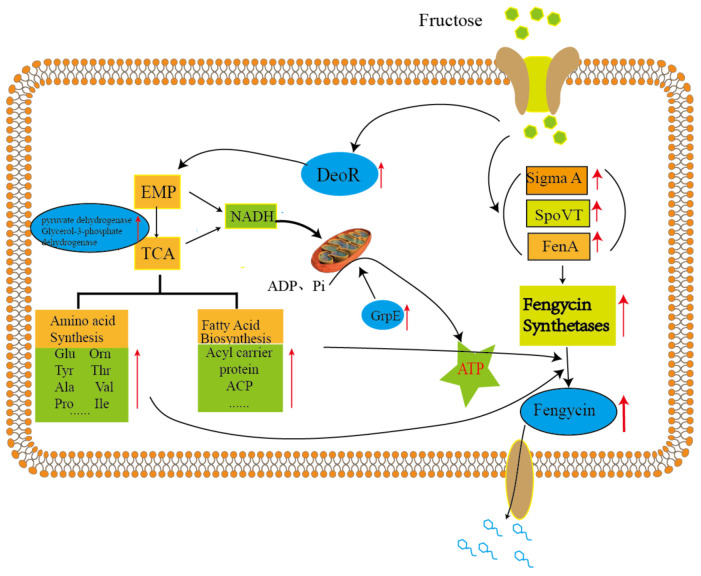
Schematic presentation of pathways of *B. amyloliquefaciens* fmb-60, which was cultivated in MLF medium is illustrated above. The identified proteins involved in nine different pathways were marked with red (up-regulation) arrows.

These alterations in protein abundance encompassed seven proteins associated with drug and toxin metabolic process that showed significant changes in expression patterns. Six include oleandomycin glycosyltransferase, adenylyl-sulfate kinase, cypemycin methyltransferase, phosphinothricin acetyltransferase, fengycin synthetase A, polyketide synthase, were upregulated, and one of them, namely Surfactin synthetase A was downregulated (Table S1). qRT-PCR results showed that the gene expressions of Fengycin synthetase A gene (*fenA*) expression was upregulated (Figure 3); this finding confirmed the previous proteomic analysis. When *B. amyloliquefaciens* cells were exposed to fructose, fengycin production was increased significantly (Figure 4). These data sustain the participation of fructose in improving the fengycin production in *B. amyloliquefaciens* cells through enhancing the pathways of fengycin biosynthesis. 

#### 2.3.7. Vitamin Metabolic Process

Vitamin is a vital molecule for the normal growth and development of most living organisms. Three upregulated proteins, including Thiazole synthase, 2-dehydropantoate 2-reductase and 3-methyl-2-oxobutanoate hydroxymethyl transferase, and one downregulated protein, Riboflavin biosynthesis protein that are associated with vitamin metabolic process in *B. amyloliquefaciens* cells exposed to fructose were identified (Table S1). qRT-PCR analysis suggested that Thioredoxin reductase gene (*trxB*) expression is both upregulated (Figure 3), according to the proteomic analysis. It was indicated that fructose was possibly improving vitamin biosynthesis, which led to the high yield of fengycin.

#### 2.3.8. Nucleobase-Containing Compound Metabolic Process and DNA Transcription and Translation

23 differentially expressed proteins, which were closely related to the Nucleobase-containing compound metabolic process and DNA transcription and translation, were identified, and eighteen of them were upregulated, while five of them were downregulated. For instance, the proteins play a vital role in DNA transcription and translation, including DeoR family transcriptional regulator, sporulation kinase, RNA polymerase sigma factor SigA, AbrB family transcriptional regulator, stage V sporulation protein T, Nucleoside diphosphate kinase and so on were upregulated. qRT-PCR results displayed that AbrB family transcriptional regulator, stage V sporulation protein T gene (*spoVT*) expressions were upregulated, which was according to the proteome analysis results (Figure 3). The findings imply that fructose may enhance fengycin biosynthesis by upregulating the proteins involved in the nucleobase-containing compound metabolic process and DNA transcription and translation in *B. amyloliquefaciens.*

#### 2.3.9. Response to Stimulus

Among the differentially expressed proteins, six proteins which was in response to stimulus (five upregulated and one downregulated) were identified. These upregulated proteins are Thioredoxin reductase, Flagellar motor switch protein FliG, protein-arginine kinase, chemical-damaging agent resistance protein C and HTH-type transcriptional repressor NsrR. One downregulated protein is Flagellar motor switch protein FliN (Table S1). qRT-PCR assays suggested that thioredoxin reductase gene (*trxB*), flagellar motor switch protein FliG gene (*fliG*) and chemical-damaging agent resistance protein C gene (*terD*) expressions were upregulated, which is in accord with the proteomic analysis (Figure 3). The results indicated that fructose is possibly involved in enhancing the response activities to the stimulus of *B. amyloliquefaciens*, such as chemical-damaging agent resistance and swimming motility. 

## 3. Discussion

Fengycin is a promising antifungal lipopeptide from *Bacillus* spp. synthesized by non-ribosomal peptide synthetases (NRPS), due to its strong, and widespread antifungal activity [16]. The antimicrobial actions of fengycin against fungi can be summarized as the destruction of cell membrane structure [17], disruption of bacterial quorum sensing system [18] and promotion of cell apoptosis [19]. Therefore, it displays tremendous application prospects in the defense against plant pathogens, such as *Fusarium oxysporum*, *Colletrotrichum*, *Alternaria alternata*, *Gloeosporioides* and *Fusarium solani* [20]. Although fengycin displays the potential to be developed as a novel antifungal agent, the poor yield is the main limitation. In our previous works, it was found that *B. amyloliquefaciens* fmb-60 is able to enhance the yield of fengycin, and the gene expression of *fen A* is upregulated in modified Landy medium added with fructose (MLF) [15], which provides a feasible strategy to increase the production of fengycin. Based on the results presented in this study of iTRAQ-based proteomic analysis and quantitative real-time PCR verification, the fructose action mode in *B. amyloliquefaciens* includes all kinds of the pathways mentioned above, such as enhanced energy metabolism, cellular amino acid, amide, peptide and fatty acid metabolic and transport process, response to stimulus and, particularly, the improvement of biological metabolism of nucleic acids. These proteins are analyzed as the following.

Previous publications have shown that catabolite repression in *Bacillus subtilis* involves transcriptional repression mediated by a negative-control system [21]. Expression of catabolite repression of *dra–nupC–pdp* operon submits to repression by glucose in *Bacillus subtilis*. It was found that the catabolite repression of *dra–nupC–pdp* operon is absent when the expression is repressed by DeoR repressor [22]. This suggests that DeoR, like glucose, inhibits the *dra–nupC–pdp* operon in *Bacillus subtilis*. In addition, three components downstream of the *dra–nupC–pdp* operon have been identified, especially the second trans-acting factor HPr, which is a component of the phosphoenolpyruvate–carbohydrate phosphotransferase system [23]. This may also account for the high content of DeoR protein in the fructose group, which promotes the metabolism process of fructose by accelerating the glycolysis process.

Energy metabolism can achieve high titer, yield and productivity (TYP) of engineered strains [24]. It was found that fructose had a significant impact on the energy state of the cell. The TCA cycle is the final common metabolism pathway for carbohydrates, fats and amino acids [25]. As one of the most important enzymes in the TCA cycle, the pyruvate dehydrogenase encoded by pdhA catalyzes pyruvate to acetyl-CoA along with the conversion of NAD^+^ to NADH. The expression of pdhA was upregulated 2.5 times in MLF. It is important in the TCA cycle because it involves the supply of NADH for the subsequent respiratory chain and promotes the TCA cycle. Glycerol-3-phosphate dehydrogenase encoded by *gspA*, upregulated 1.5 times in MLF, which interconverted with dihydroxyacetone phosphate by glycerol-3-phosphate dehydrogenases provides a link between carbohydrate and lipid metabolism [26]. Those two enzymes are important in the energy supply and carbon metabolism cycle, which have a great function on amino acids from fengycin biosynthesis and the assembly of NRPSs system. In addition, MLF has a higher reduction state and greater production of GrpE in fermentation compared with ML medium. GrpE, located in bacterial cytoplasm, plays a prominent function in promoting the exchange of ADP for adenosine triphosphate (ATP) [26]. Promoted glycolysis and the tricarboxylic acid cycle, as well as oxidative phosphorylation, provide a carbon skeleton for the biosynthesis of amino acids [15].

Lipid structure plays an essential role in the structure and function of fengycin. Hence, the fatty acid biosynthesis system is primarily for the synthesis of fengycin, especially for branched-chain fatty acids. β-ketoacyl-acyl carrier protein synthase catalyzes the condensation of a malonyl-acyl carrier protein (ACP) with acetyl-CoA to form β-ketobutyryl-ACP, which is the first step of straight-chain saturated fatty acid biosynthesis [27]. The branched-chain fatty acid synthesis precursors such as isobutyryl-CoA, isovaleryl-CoA and α-methylbutyryl-CoA can be derived from the branched-chain amino acids including L-valine, L-leucine, and L-isoleucine, respectively. Thus, the biosynthesis of branched-chain amino acids is an essential component for fengycin biosynthesis. Moreover, the biosynthesis of L-isoleucine, L-valine and L-leucine are catalyzed by the same enzyme system encoded by *ilvBN, ilvGM, ilvIH, ilvC, ilvD*, and *ilvE* [28,29]. In our study, *ilvB* expression was found up-regulated by a factor of 1.5, suggesting that fructose can induce biosynthesis of branched-chain amino acids, thereby promoting the accumulation of fengycin production. Other small molecules, such as vitamins, were also increased, which may be because they act as modulators or enzyme auxiliary groups to promote the biosynthesis of precursors fengycin.

Fengycin is synthesized by a non-ribosomal pathway; therefore, the biosynthesis process of fengycin is hardly affected by the gene transcription process, but the gene transcription of fengycin synthase is tightly regulated by the gene regulatory network. Previous studies it was suggested that endogenous factors like two-component regulatory factors (ComA/ComP), sigma factors and signal proteins (DegU, DegQ and PhrC, AbrB) display an indispensable role in fengycin synthetase genes expression [30]. Sigma factor is a transcriptional regulator that can identify mRNA transcription start sites and promote gene expression among them. Findings from the proteomic analysis released that RNA polymerase sigma factor SigA up-regulated content. Conversely, the signal protein AbrB is a general transcriptional repressor of lipopeptide synthetase [31], AbrB family transcriptional regulator, stage V sporulation protein T gene (*spoVT*) was found to be upregulated significantly in the supplemental fructose group, which indicates that fructose can promote the transcription and expression of fengycin synthase gene. In addition, it was revealed that in MLF, *B. amyloliquefaciens* fmb-60 is able to express a higher level of enzymes related to protein transport and modification, such as phosphoserine phosphatase, which can promote the transformation of the original peptide into mature polypeptide units, that may also response to increase fengycin production.

## 4. Materials and Methods

### 4.1. Microorganisms and Fermentation

*B. amyloliquefaciens* fmb-60 (CGMCC 7.222), which can produce fengycin, was inoculated in 50 mL Landy medium (5 g/L sodium chloride, 5 g/L beef extract, 5 g/L yeast extract, 10 g/L tryptone, 10 g/L glucose) and cultivated in a rotatory shaker at 37 °C and 180 rpm for 10 h as the initial seed culture. Then, 5% (*v/v*) seed culture was inoculated into 100 mL modified Landy (ML) medium (1 g/L yeast extracts, 14 g/L L-Glutamate sodium, 0.5 g/L KCl, 1 g/L KH_2_PO_4_, 0.5 g/L MgSO_4_, 5 mg/L MnSO_4_, 0.16 mg/L CuSO_4_, 0.15 mg/L FeSO_4_ •7H_2_O) and 100 mL ML medium was added with fructose, named as MLF medium. They were then incubated on a rotary shaker (180 rpm) at 33 °C for 72 h. Finally, the fengycin in the fermentation cultures were determined following the previously described method. Moreover, the antimicrobial activity of the fermentation cultures against *Aspergillus flavus* were conducted through inhibiting zones test.

### 4.2. Preparation Sample Preparation and LC-MS/MS Analysis

The proteins were extracted from samples of ML and MLF, frozen in liquid nitrogen, and then the bacteria cells were collected by centrifugation at 8000 g for 10 min at 4 °C and washed 3 times with cold PBS buffer (0.01 M, pH 7.2, 4 °C). After that, the bacteria were placed in a clean mortar, ground with liquid nitrogen for 20 min until powdered, transferred to an ultrasonic crushing tube, and protein extract (7 M urea, 2 M thiourea, 4% CHAPS, 1% DTT, and 10 mM protease inhibitor) was added. After breaking the cells by ultrasound for 10 min, the supernatant was collected and added with acetone with a ratio of 1:3 (*v/v*) after acetone was cooled at −20 °C, precipitated for 12 h at −20 °C, and centrifuged at 14,000 g for 30 min at 4 °C. The protein precipitate was washed twice by using cooled 80% aqueous acetone solution, placed on a sterile operating table, air-dried, the residual acetone was removed, 0.5 mL of UA buffer (8 M urea, 0.1 M Tris/HCl, pH 8.5) was added and redissolved [32].

The protein extract was quantified using Microplate BCATM Protein Assay Kit-Reducing Agent Compatible (Beyotime, P0010, Jiangsu, China) with bovine serum albumin as a standard. A certain extract volume containing 200 μg protein was placed in an ultrafiltration tube (Amicon Ultra-0.5 Filter (Millipore). After DTT reduction, iodoacetamide alkylation, and trypsin hydrolysis for 16 h, the extract was dried by rotary vacuum concentrator, and 0.2% formic acid solution was added for peptide quantification by micro nucleic acid quantification. The groups were labeled following the iTRAQ 8-plex kit instructions, markers 115 and 116 and 117 were labeled the control group (ML), and markers 118 and 119 and 121 were labeled the treatment group (MLF).

After labeling, the four groups of samples were mixed with a 2 mL Enffendof tube and dried in a rotary vacuum concentrator. The iTRAQ labeled peptide mixture was dissolved using 100 μL of high pH RP buffer A (98% ultrapure water, 2% methanol, pH 10.0), and the iTRAQ labeled peptides were analyzed using an ACQUITY UPLC BEH C18 via a U3000 HPLC system at a flow rate of 0.2 mL/min, and eluted by a program: 3% buffer B (2% ultrapure water, 98% methanol and pH 10.0) for 12 min; 3–18% buffer B for 30 min; then 18–32% buffer B for 14 min; then 32–98% buffer B for 10 min; and finally, 97% buffer A for 10 min. The eluted fractions were measured at 214 nm and 280 nm, and interval 200 μL was collected at 1 min. In total, 60 tubes were collected, and 5 tubes were combined into one tube after random sorting, and the samples were divided into 12 tubes and dried by rotary vacuum concentrator. The graded peptides were re-solubilized in the loading buffer (2% acetonitrile, 0.1% trifluoroacetic acid and ultrapure water) and analyzed by NanoLC-nanoESI-MS/MS using an Acclaim PepMap100 C18.

The raw data obtained from mass spectrometry analysis were analyzed by Proteome Discoverer 1.4 system and compared with the *B. amyloliolyticus* proteome database by SEQUEST search engine for protein identification. The selected database was obtained by downloading from the Uniprot online database in July 2021. The database contains a total of 86,716 *B. amyloliquefaciens* protein information. Proteins were considered significantly different only if the number of protein changes was larger than 2 and at least one specific peptide was identified. Protein functional interpretation was carried out by using the Uniprot online website (http://www.uniprot.org/ (accessed on 11 October 2021)), and protein GO clustering analysis was performed by using the Protein Information Resource (http://pir.georgetown.edu/ (accessed on 11 October 2021)) with GenomeNET online website (http://www.genome.jp/ (accessed on 11 October 2021)).

### 4.3. Extraction of RNA and the Quantitative Real-Time PCR (qRT-PCR)

The total RNA extraction was conducted using the Trizol method (Invitrogen Life Technologies, Inc., Gaithersburg, MD, USA) following the instructions. The concentration and purity of total RNA were determined by Nano Drop 2000. The extracted total RNA was used as a template to synthesize the cDNA first strand using a HiScript First Strand cDNA Synthesis Kit (Vazyme, Nanjing, China). The qPCR reaction system was performed according to TaKaRa SYBR Premix ExTaqTMII kit, after the addition of primers (Table 1) and 1 ng cDNA template being added to the reaction system, the reaction system was conducted under the following settings: (1) denaturation at 95 °C for 30 s, (2) denaturation at 95 °C for 5 s, extension at 60 °C for 30 s, and cycling for 40 times. The fluorescence signal was collected at 60 °C extensions in this stage, and (3) denaturation at 95 °C for 15 s, extension at 60 °C for 60 s, and denaturation at 95 °C for 15 s, then the dissolution curve was detected. The analysis of relative expression level of target genes was performed in triplicate.

### 4.4. Statistical Analysis

Student’s test was performed for comparison of only two groups and one-way ANOVA for comparison multiple group comparison (*p* < 0.05).

## 5. Conclusions

Fengycin is not able to be obtained in large quantities due to low biosynthesis in *B. amyloliquefaciens* fmb-60, which limits its development and application in the preservation of agricultural products. Based on a detailed understanding of the high-yield fengycin biosynthesis mechanism, optimizing the fermentation process is an effective strategy to increase the yield of fengycin. According to the proteome analysis, the mechanism of fructose enhancing fengycin biosynthesis in *B. amyloliquefaciens* fmb-60 can be summarized as improvement of the metabolic process including cellular amino acid and amide, fatty acid biosynthesis, peptide and protein, nucleotide and nucleobase-containing compound, drug/toxin, cofactor, and vitamin; reinforcement of peptide/protein translation, modification, biological process and response to a stimulus. The results can help understand the mechanism of fructose on improving fengycin biosynthesis, which will provide the theoretical basis for improving the yield of fengycin or its analogs in the fermentation industry, and applying the antimicrobials to resist filamentous fungi in the food and feed industries.

## Figures and Tables

**Table 1:** Primers used for quantitative RT-PCR Analysis for selected genes.

No	Protein ID	Protein Name	Gene	Primers
1	I2C429_BACAM	Gamma-glutamyl phosphate reductase	*proA*	F-GCGCTCGTCAGTGTCATTTA
				R-GGTTTGGCTGTTTCGTCAAT
2	I2C0D4_BACAM	Serine--tRNA ligase	*serS*	F-AACCTGCCGATCAATTATGC
				R-ATCACCGGTACACATGCTCA
3	A0A0D7XLI1_BACAM	50S ribosomal protein L23	*rplW*	F-TAAGCGCCCCGTCATTACT
				R-TTCTGCGACGGCTAGTCATA
4	A0A0D7XV55_BACAM	30S ribosomal protein S4	*rpsD*	F-ATTCCGCACATTGTTTGACA
				R-CCGATTGTTTGACCAGGTTT
5	I2C4J1_BACAM	Pyruvate dehydrogenase E1 component subunit alpha	*pdhA*	F-CGCTTGAACAGGACGACTTT
				R-GTCTGTGCGGATGATTGCTT
6	I2C7G5_BACAM	Glucose-6-phosphate 1-dehydrogenase	*zwf*	F-AGTCATCCGGTTTGCGAATG
				R-TTCGCTGCGGATTTCTTCTG
7	I2C738_BACAM	Glycerol-3-phosphate dehydrogenase [NAD(P)+]	*gpsA*	F-ACAAAAGGGGTCCATCTCGT
				R-CAGCCATTTTACGGTAGCCC
8	I2C8P2_BACAM	Long-chain fatty-acid-CoA ligase	*lcfA*	F-TGAGGAGCTGGCAGGTAAAA
				R-GGTATGACGACAATTCCCGC
9	I2C8L1_BACAM	Acetolactate synthase	*ilvB*	F-ACTGGTTTTATGCCGTCACG
				R-CCCTGAAGGCGTTTGTTACC
10	CHEY_BACSU	Chemotaxis protein CheY	*CheY*	F-GGAAGCTGAAAATGGAGCAC
				R-TGATTGCTGTCCCATAGCAG
11	H9TE70_BACAM	Fengycin synthetase A	*fen A*	F-GGAATCACAGAGCAGCATGA
				R-TAAGCAGATGGTCGGCTTCT
12	I2C3L7_BACAM	Thiazole synthase	*thiG*	F-AAAGCTTCAGGGCTTTGTGA
				R-CACTCCGAGCTCCTCAAGAC
13	I2C0H9_BACAM	AbrB family transcriptional regulator, stage V sporulation protein T	*spoVT*	F-GTGACGCGTATATTGCCGTT
				R-GAAAAGATGACCACGGCACC
14	TRXB_BACSU	Thioredoxin reductase	*trxB*	F-AACAGGCTGCGACATTTGAG
				R-CCGGAAATCACGACATGCTT
15	A0A1Y0X6E7_BACAM	Chemical-damaging agent resistance protein C	*terD*	F-ATGGTGCTTGTCATGAACGG
				R-TCTGTATCCGCGTCCATCTC
16	I2C550_BACAM	flagellar motor switch protein FliG	*fliG*	F-TAAATTACGCCCGCCAAGTG
				R-TGCGTAAAGGAGGAGGACAG
17	Housekeeping gene		*gatB_Yqey*	F-AGCTGGTCGTGAAGACCTTG
				R-CGGCATAACAGCAGTCATCA

The sequences of target genes were obtained from NCBI, and the primers were designed by Primer3web online.

## Data Availability

Not applicable.

## Supplementary Materials

The following are available online, Table S1: protein fold-change.
